# Anterior minimally invasive bridge-plate technique for treatment of humeral shaft nonunion

**DOI:** 10.1007/s10195-012-0203-1

**Published:** 2012-06-21

**Authors:** Paulo Roberto Vilaça, Marcelo Koh Uezumi

**Affiliations:** Brazilian Orthopaedics and Traumatology Society—SBOT, Associação Beneficente Nossa Senhora do Pari, Hospital Nossa Senhora do Pari, Rua Hannemann, 234, São Paulo, SP Brazil

**Keywords:** Nonunion, Humerus, Bridge plate, MIPO

## Abstract

**Background:**

The present study introduces a new surgical technique and the results of a case series of patients with humeral shaft nonunion.

**Materials and methods:**

Fifteen patients with diagnosis of diaphyseal nonunion of humerus were operated by a bridge-plate technique. A 4.5-mm plate is slid on the anterior surface of the humerus, submuscular to the brachial muscle. With the plate over the anterior surface of the humerus, screws are inserted from anterior to posterior on the ends of the plate. When there is a small bone gap, an iliac autologous graft is inserted. Minimum follow-up was 1 year.

**Results:**

Bone healing was obtained in all patients: 1.5 months postoperatively in 11 patients, 2 months in 3 patients, and 3 months in 1 patient. There were no postoperative infections, there was one case with loosening of the screws and plate, and there were no nerve injuries.

**Conclusions:**

The present technique avoids wide dissection, radial nerve isolation, and periosteum stripping. The anterior minimally invasive bridge-plate technique for treatment of humeral shaft nonunion is a safe procedure and obtained bone healing in all patients in this series.

## Introduction

Humeral nonunion is a condition resulting from lack of healing at the fracture site often associated with alteration of the local biological potential. Due to the preoperative conditions and the difficulties inherent to the surgery, treatment is a challenge.

Ring et al. [10] demonstrated that the most important factor to achieve bone healing in nonunion is use of a careful, biologically and mechanically adequate technique.

Using a surgical technique that respects the basic principles of less soft tissue dissection, preservation of blood supply, and immediate rehabilitation of the operated limb, the present study introduces a new surgical technique for treatment of humeral shaft nonunion and the results of a case series of patients.

## Materials and methods

Fifteen patients (6 women and 9 men) with diagnosis of diaphyseal nonunion of humerus were operated on by the same surgeon between July 2008 and June 2010.

The Regional Ethics Committee approved this study (protocol number CAAE: 254.0.162.000-10), and all subjects signed informed consent. The study has been performed in accordance with the ethical standards laid down in the 1964 Declaration of Helsinki.

Mean patient age was 51.5 ± 21.1 years (range 23–85 years) with nonunion for an average of 11.5 ± 5.4 months (range 6–24 months). Seven patients had undergone previous surgery after the initial trauma. Of these, four had already been reoperated for the established nonunion, unsuccessfully receiving plates and screws with autologous bone grafting in this second operation (Table 1).

**Table 1:** Preoperative data

Patient	Age (years)	Type	Previous treatment	Nonunion duration (months)	Complaint
1	54/M	Hypertrophic	Conservative	9	Instability
2	62/M	Atrophic	Plate fixation	12	Instability
3	54/M	Atrophic	Plate fixation	14	Instability
4	35/M	Atrophic	Nail	6	Pain
5	29/M	Atrophic	Plate fixation	24	Pain
6	68/F	Atrophic	Conservative	6	Pain + instability
7	26/M	Atrophic	Nail	12	Pain + instability
8	85/F	Atrophic	Conservative	12	Pain + instability
9	24/F	Hypertrophic	Nail	6	Pain
10	36/M	Hypertrophic	Conservative	6	Pain
11	71/M	Atrophic	Conservative	12	Pain + instability
12	73/F	Atrophic	Conservative	12	Instability
13	77/F	Atrophic	Plate fixation	18	Pain + instability
14	55/M	Atrophic	Conservative	6	Pain + instability
15	23/F	Atrophic	Plate fixation	18	Instability

The inclusion criterion was diaphyseal nonunion of humerus. Cases with active infection were excluded. Nonunion was defined as lack of bone healing at least 6 months after the fracture [3]. The type of nonunion was classified by radiographic standards [3] as being atrophic or hypertrophic. In this series, 12 cases were atrophic and 3 were hypertrophic.

Four patients presented with osteoporotic bones. In these patients, no good screw fixation could be achieved so a locking plate (LCP) and screws were used.

Clinical and radiographic evaluations were done 0.5, 2, 3, 6, and 12 months postoperatively. After this period, follow-ups were done every 6 months. University of California, Los Angeles (UCLA) score system [2] was adopted for shoulder function clinical evaluation. The bone healing criteria were clinical absence of pain and mobility, and radiographic presence of healed cortices.

### Statistical analysis

A related-sample Wilcoxon signed-rank test was used to compare pre- and postoperative UCLA scale values and pre- and postoperative elbow function. Data are presented as average ± standard deviation (SD). Statistical significance was set at 0.05.

Minimum follow-up was 1 year with a range of 1–3.5 years.

### Operative technique

The patient is operated in supine position, with the bone graft removed from the contralateral iliac crest in case of atrophic nonunion.

In the distal humerus region, a 4-cm anterior longitudinal incision is made on the lateral border of the biceps. Between the biceps and the brachioradialis muscles, the brachial muscle can be accessed more deeply. A blunt longitudinal midline opening is made in the fibers of the brachial muscle. The brachialis muscle is innervated at the lateral surface by the radial nerve and medially by the musculocutaneous nerve. This anatomical detail allows its longitudinal opening without any harm to nerves or loss of function; then, access to the anterior surface of the humerus is easily achieved. Through this opening, the screws will be fixed distally.

**Fig. 1:**
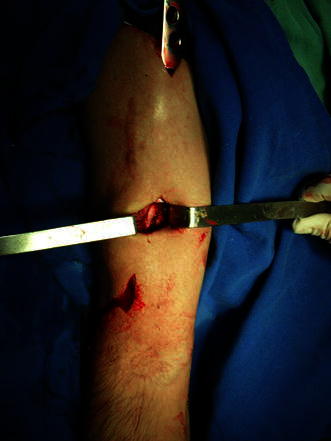
With the plate slid in over the anterior surface of the humerus, the screws are inserted from anterior to posterior on the extremities of the plate. Through an auxiliary incision on the anterior surface of the humerus, the bone graft is inserted into the atrophic nonunion site. Distally the radial nerve is not seen and is protected laterally by muscles

Distally, the muscles including the lateral part of the brachialis and the brachioradialis protect the radial nerve, which is neither seen nor dissected in this approach. Proximally, a 4-cm incision is made between the medial border of the deltoid and the lateral border of the biceps. The interval between these two muscles is the location to slide in and fix the plate. A 4.5-mm dynamic compression plate (DCP) (or LCP in osteoporotic cases) must be slid on the anterior surface of the humerus, submuscular to the brachial muscle [7] (Fig. 1). The direction in which the plate is slid differs according to each case. Distally, one must be careful not to violate the coronoid fossa.

**Fig. 2:**
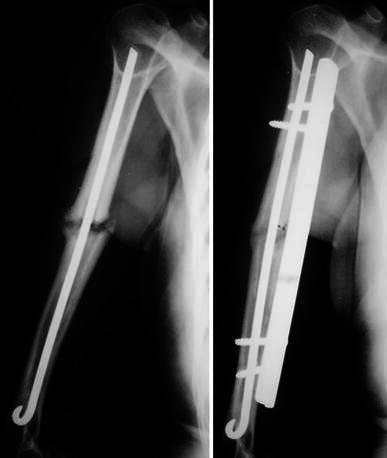
Hypertrophic nonunion case. The plate is passed and fixed over the anterior surface of the humerus. The Rush nail was maintained because there was no complaint and it would be difficult to remove without bone aggression

With the plate slid over the anterior surface of the humerus, the screws (two or three in each fragment normally) are inserted from anterior to posterior on the ends of the plate. In case of hypertrophic nonunion, no further surgery is needed (Fig. 2).

In case of atrophic nonunion, a 3-cm auxiliary incision is made over the nonunion site on the anterior surface of the humerus with direct dissection to the site. Fibrous tissue is removed, and the bone prepared. With a small bone gap (less than 1 cm), a cancellous iliac autologous graft is inserted. With greater bone loss, the structured bone graft is “press-fitted” into the nonunion without the need for complementary fixation.

### Rehabilitation

Immediately postoperatively, patients were directed to use the limb in their usual activities, and to use a sling only for pain control in the first 5 days if necessary, mainly at night when sleeping. Use of immobilization was not advised after this period.

All patients were recommended to move the elbow and shoulder, avoiding stiffness.

After bone healing, a rehabilitation program was performed. The first aim was to gain full mobility, then proprioception, and finally muscular strengthening. The total rehabilitation period depends on the progression of each patient. The final goal is to restore full range of motion and strength without pain.

## Results

Bone healing was obtained in all patients: after 1.5 months postoperatively in 11 patients, 2 months in 3 patients, and 3 months in 1 patient (Table 2). There were no postoperative infections. There was one case with loosening of the screws and plate. There were no nerve injuries.

**Table 2:** Postoperative data

Patient	Time to union (weeks)	Shoulder function (UCLA)/Ellman criteria	Elbow function extension/flexion (°)	Final complaint
1	6	35/excellent	0/135	None
2	6	33/good	10/122	None
3	8	25/satisfactory	40/110	Iliac crest pain
4	12	27/satisfactory	30/120	None
5	6	33/good	22/130	Iliac crest pain
6	6	27/satisfactory	38/114	Iliac crest pain
7	6	35/excellent	0/110	None
8	6	22/satisfactory	30/100	None
9	6	35/excellent	5/130	None
10	6	27/satisfactory	8/130	None
11	6	32/good	0/130	Iliac crest pain
12	6	26/satisfactory	16/135	Iliac crest pain
13	8	29/good	40/110	None
14	8	19/poor	18/128	Diffuse pain on arm
15	6	35/excellent	0/128	None

The case with hardware loosening was reoperated using the same bridge-plate technique. Plate and screws were removed, better reduction was performed, and new bone graft applied. Bone healing was obtained in 2 months. As better reduction was achieved and there was good fixation of the screws, immobilization was used postoperatively for 5 days only and the rehabilitation protocol described above was used.

Twelve cases received autologous iliac crest bone graft. There were only slight symptoms at the bone graft donor site in five patients.

The average UCLA score [2] of shoulder function was 18.4 ± 6.7 (range 10–28) preoperatively and 29.3 ± 5.1 (range 19–35) postoperatively, representing a statistically significant improvement (*p* ≤ 0.001) (Table 2).

Preoperatively, based on the UCLA score and the Ellman classification [2], two patients had good function, five were rated as satisfactory, and eight as poor. Postoperatively, four patients had excellent function of the shoulder, four had good function, six were rated as satisfactory, and one as poor. This was a case of malunion of a previous proximal humerus fracture.

After 3 months, four patients were asymptomatic with normal function of the operated limb.

The lack of elbow extension averaged 32.5 ± 20.9° (range 0–80°) preoperatively and 17.1 ± 15.3° (range 0–40°) postoperatively, representing a statistically significant improvement (*p* = 0.003). Elbow flexion averaged 110.5 ± 13.9° (range 80–130°) preoperatively and 122.1 ± 10.9° (range 100–135°) postoperatively, representing a statistically significant improvement (*p* = 0.001) (Table 2).

## Discussion

This case series shows that functional results after humeral shaft nonunion can be obtained with a minimally invasive approach and without bone graft in hypertrophic nonunions. This technique has certain advantages that make it appealing to the trauma surgeon.

Plates can be safely used anteriorly along the humerus with this technique. Good results have been achieved with submuscular plating with no major soft tissue problems and with functional results similar to other methods in the literature. Previous studies demonstrated faster recovery with minimally invasive techniques compared with invasive open technique [5, 7, 8].

The healing of the humeral shaft fractures in this series presents good results with the advantage of using a minimally invasive technique. This fixation aims at maintaining bone alignment through indirect reduction without an open approach to the fracture site. This preserves the local blood supply and results in less surgical damage to soft tissues, replacing absolute stability by relative stability to achieve bone healing by stimulating bone formation.

There are several studies in the literature showing the advantages of using a bridge-plate in fractures of different parts of the body, such as the femur and the tibia, but there are few reports of use of this technique for humerus [7]. Also, there is a lack of reports in the literature on use of this technique for nonunions.

Being a minimally invasive technique, complications are reduced [7, 8]. There were no nerve injuries. The bridge-plate technique for treatment of humeral shaft nonunion is indicated for cases of both atrophic and hypertrophic nonunion.

A limitation of this study is the inhomogeneous patient population. There is a large age range (23–85 years) with different types of nonunion, and some patients with osteoporotic bones. The objective of the present study is not to compare techniques or healing times between atrophic or hypertrophic nonunions, but rather only to demonstrate that it is possible to use a minimally invasive technique for nonunion and present the results as an alternative to the traditional technique. The lack of a homogeneous population does not influence the results.

**Fig. 3:**
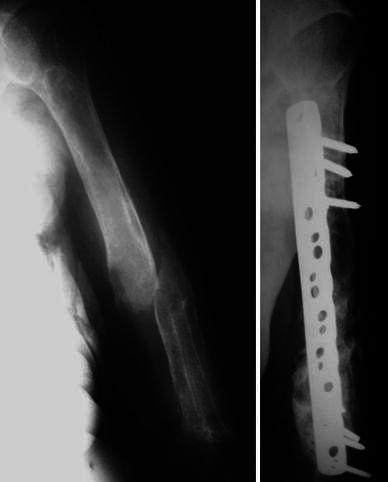
Atrophic nonunion in an osteoporotic bone (*left*). After cleaning and grafting focus and bridge-plate fixation with locking plate and screws, the nonunion is healed (*right*)

Use of different plates (DCP or LCP) did not influence the results. Locking screws were used in osteoporotic bones as an alternative for better bone fixation (Fig. 3). The space between distal and proximal fixation was the same as for the DCP fixed with cortical screws. Relative stability was present in both situations. Future studies should compare the two techniques and show their specific indications. Results show that the minimally invasive technique is versatile and can be used with good results for atrophic and hypertrophic nonunions.

It is shown herein that there is a biological capacity for humeral shaft healing achieved through the bridge-plate technique, with use of bone grafting in atrophic cases. It is not necessary to have absolute stability or focal compression, contradicting previous works [5].

Without the need for broad dissection, the local blood supply is preserved. By combining the biological stimulus promoted by the bone graft and the sufficient mechanical stability granted by the plate, all elements necessary for healing of nonunion are present [6].

**Fig. 4:**
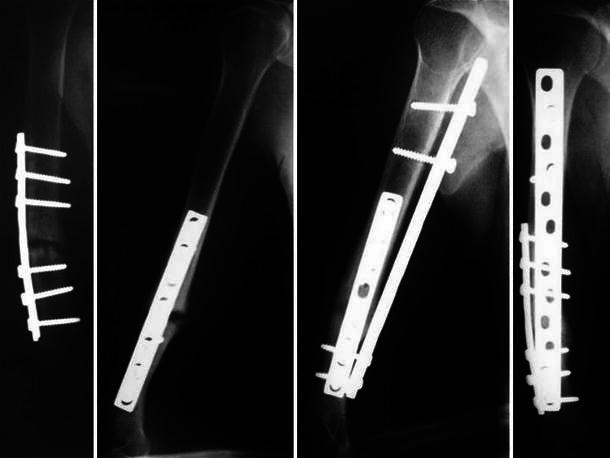
X-ray images (frontal and lateral views) demonstrate atrophic nonunion instability signs—varus alignment and initial plate bending (*left*). Six weeks postoperatively with bone formation in the nonunion site (*right*)

**Fig. 5:**
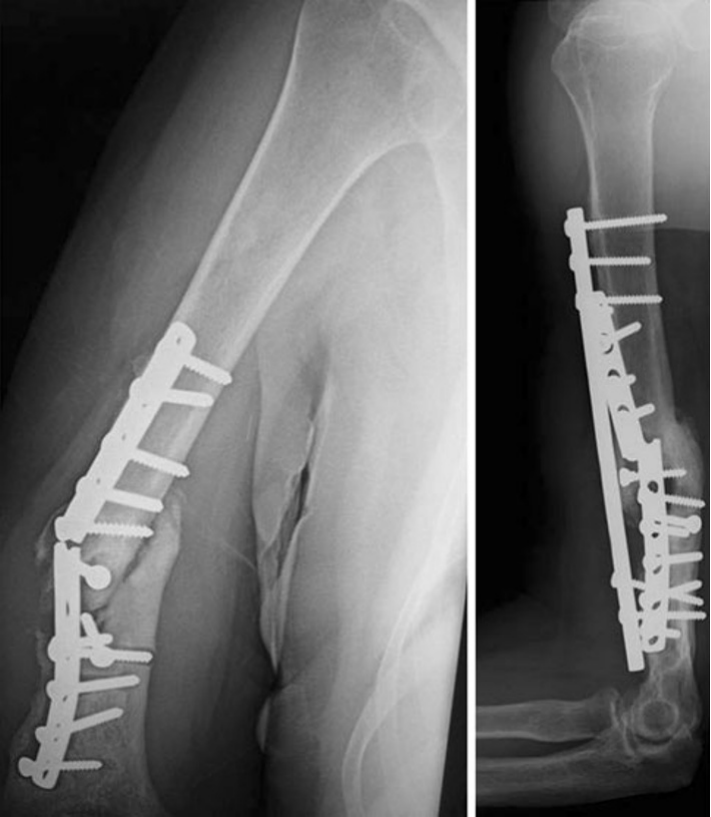
X-ray images demonstrate atrophic nonunion with previous instability signs (*left*) healed 6 weeks after the surgery (*right*)

Following the principle of minimizing invasiveness and according to previous reports [4, 8, 9], the fixation material was not removed in previously operated patients (Figs. 2, 4, 5).

The present technique makes a difficult procedure much easier by avoiding wide dissection, radial nerve isolation, and periosteum stripping.

The results obtained confirm the conclusions of Ring et al. [10], who stated that to achieve bone healing it is important to use a careful, biologically and mechanically adequate technique.

With the plate slid in over the anterior surface of the humerus and respecting the approaches previously described, the radial nerve is totally protected [1, 7] and there is no inherent danger to any vascular structure.

It is important, in future studies, to identify the limitations of the technique and define if it is applicable for more severe cases, as well as whether there are possible alternatives to the use of autologous bone graft.

In conclusion, this series demonstrates that the anterior minimally invasive bridge-plate technique for treatment of humeral shaft nonunion presents satisfactory results with regards to bone healing and functional capacity.
